# Depression, Anxiety, and Suicide Among Adolescents: Sex Differences and Future Perspectives

**DOI:** 10.3390/jcm14103446

**Published:** 2025-05-15

**Authors:** Giovana V. R. Toro, Paula Arias, Alejandro de la Torre-Luque, Jonathan B. Singer, Natalia Lagunas

**Affiliations:** 1 Department of Legal Medicine, Psychiatry and Pathology, School of Medicine, Complutense University of Madrid, Ciudad Universitaria, 28040 Madrid, Spain; gvidotto@ucm.es (G.V.R.T.);; 2 School of Social Work, Loyola University Chicago, Maguire Hall, 5th Floor, 820 North Michigan Avenue, Chicago, IL 60611, USA; jsinger1@luc.edu

**Keywords:** depression, anxiety, suicide, adolescence, sex differences

## Abstract

Depression and anxiety are two disorders that significantly increase the risk of suicidal behavior. The disparity between females and males in the prevalence of these disorders becomes more pronounced from adolescence onwards. Specifically, risk factors associated with suicidal behavior in adolescent females are often linked to anxiety and depression, whereas in males, these behaviors are more frequently associated with other types of disorders, such as disruptive behavior. Furthermore, there are notable sex differences in the efficacy and acceptance of treatments aimed at preventing suicidal behavior. This review examines the sex differences in the relationship between depression, anxiety disorders, and suicidal risk in adolescents. Specifically, it aims to identify key risk factors influencing suicide vulnerability across sexes and assess the efficacy of current treatment approaches in mitigating these sex specific risks.

## 1. Introduction

In 2019, nearly one billion people suffered from a mental disorder, and the two most frequent were anxiety and depression [1]. Understanding this global burden requires examining the multifaceted factors that influence mental health across populations. Well-being is a product of the complex interrelation among biological, psychological, and sociocultural determinants, each playing a crucial role in shaping both vulnerability and resilience [2]. The strong link between physical and psychological health underscores the need for greater attention and resources for mental disorders, given their often invisible impact on public health systems, social support networks, and society as a whole [3]. To address this challenge, proactive measures such as widespread mental health education in schools, early screening programs in primary care settings, improving psychological and psychiatric hospital capacities, and integrating mental health professionals into community health initiatives are mandatory. Additionally, expanding subsidized therapy and psychoeducational interventions can help strengthen prevention efforts and resilience in vulnerable population, reducing the burden on both individuals and society.

Globally, the two most common psychiatric diagnoses are depression and anxiety [4]. Depressive disorders are related to feelings of sadness, emptiness, and irritable mood that may include feeling a loss of pleasure and generalized disinterest. On the other hand, anxiety is characterized by excessive fear and concerns about future threats. Both diagnoses can significantly impact physical health and result in functional impairment. The onset of anxiety and depressive disorders is most frequently during childhood or adolescence [5,6,7].

Adolescence is a crucial stage of development defined by marked hormonal, physical, and psychological changes. This time period is also a transition stage, from childhood to adulthood, weighted by myriad expectations from both family and society, and the influence of developmental transitions and social roles [8].

Puberty begins with physical changes driven by the activation of the hypothalamic–pituitary–gonadal axis, while sex hormones influence hypothalamic–pituitary–adrenal (HPA) axis function, establishing a neuroendocrine coupling that regulates stress response [9] and contributes to sex differences in stress reactivity [10]. Increased testosterone during adolescence is linked to reduced HPA reactivity to stressors, whereas higher dehydroepiandrosterone (DHEA) levels correlate with a more adaptive stress response [11,12]. Moreover, adolescent cortisol profiles influence coping strategies. Normative cortisol patterns have been associated with the use of more adaptive coping strategies, which, in turn, correlate with more regulated cortisol responses [13,14].

During adolescence, females exhibit heightened negative affective responses to stress compared with males. These sex differences emerge during puberty and persist into adulthood, with females showing greater corticolimbic reactivity and males exhibiting higher HPA and autonomic activation [10]. Despite lower peripheral physiological responses, females report stronger emotional distress, possibly due to increased activity in corticolimbic circuitries, which are involved in translating stress into subjective awareness [10]. Additionally, hormonal fluctuations during the menstrual cycle influence stress response and coping strategies [15,16], potentially contributing to sex differences in vulnerability to emotional disorders, which will be expanded upon in the following sections.

The psychological and social challenges, along with significant developmental changes in the nervous and endocrine systems make this a high-risk developmental period for adolescents [17]. In addition to the well-established prevalence of mental illness, adolescence has also become a time in this complex context where there is growing recognition of the rising rates of suicidal thoughts and attempts among adolescents.

Suicide is the third leading cause of death among 15 to 29 year olds [18]. The scientific literature points to a significant correlation between suicide and mental health problems, especially mood disorders. In addition, major depressive episodes are also highly correlated with suicide. Depression is closely linked to both suicidal thoughts and attempts, but it lacks specificity as a reliable predictor of suicide [19,20,21,22].

Strong and consistent associations have been identified between suicidal ideation, anxiety, and the quality of adolescents’ relationships with parents and peers. Negative experiences, such as victimization, conflict, isolation, and loneliness, were particularly linked to both suicidal ideation and anxiety. A global study of adolescents across 82 countries found high prevalence rates of suicidal ideation (14.0%) and anxiety (9.0%) among 275,057 individuals aged 12–17. Africa recorded the highest suicidal ideation (21.0%) and Asia the lowest (8.0%), while anxiety peaked in the Eastern Mediterranean region (17.0%) and was least common in Europe (4.0%). These findings highlight widespread adolescent mental health concerns, irrespective of income level [23].

Given the complex nature of suicide and its relationship with symptoms of depression and anxiety, greater attention must be focused on designing possible interventions for adolescents. The scientific literature includes multiple studies suggesting various effective treatment options for adolescents. One such study performed a systematic review [24] and assessed the advantages and applications of multiple viable alternatives: individual- or family-based interventions, including psychotherapy and pharmacological interventions; community-based interventions, including creative activities (music, dance, singing, arts) to build self-esteem and self-confidence; school-based interventions, aiming to promote mental health through psychoeducation (life skills, resilience) as well as programs related to suicide prevention that included screening, gatekeeper programs, and skills training; and interventions based on mental health digital platforms. The results pointed to some evidence that group-based interventions and cognitive behavioral therapy (CBT) supported a reduction in depressive and anxiety symptoms. Likewise, school-based interventions (gatekeeper programs) were found to be effective in increasing knowledge of suicide, although changes in behavior were not detected. Some positive effects on behavioral changes were observed with the use of community interventions [25].

Many types of interventions should be considered when addressing the mental health of young people. These include individual-level interventions, such as psychotherapy and medication, as well as universal interventions involving schools and communities. A literature review indicated that different interventions yield varying outcomes. One particularly effective approach is delivering health services at schools, which was found to be more effective and preferred over clinic- or community-based interventions [26]. However, it is crucial to recognize that mental health treatments for adolescents are complex, and a holistic approach that integrates biological, psychological, and social factors may be more effective.

In light of this complex scenario, this review intends to examine the relationship between depression, anxiety, and suicide in adolescents, as well as to identify any possible differences due to sex. Finally, we will discuss promising interventions to address this serious public health issue.

## 2. Sex Differences in Depression and Anxiety During Adolescence

Approximately 14% of adolescents globally experience mental health problems; the most common disorders are anxiety and depression, and these are frequently the source of additional illness and disability among teenagers. Given their strong association with suicidal thoughts and behaviors, addressing these conditions is crucial for suicide prevention among youth [18,27].

These findings align with a systematic review and meta-analysis by the World Health Organization, which examined 72 studies published globally (Asia, North America, South America, and Oceania) from 2001 to 2020 which found elevated prevalence of Major Depressive Disorder (8%), dysthymia (4%), and depressive symptoms (34%) among adolescents aged 10 to 19 years old. Depressive symptoms were more prevalent among female adolescents than male adolescents [24].

Likewise, data from the Global Burden of Disease Study highlight the global prevalence of mental disorders among adolescents, especially anxiety and depressive disorders. Among 10- to 14-year-olds, the estimated rate for all mental disorders was 12.41%, and 13.96% for adolescents ages 15–19. Anxiety symptoms were reported in 3.35% in the younger group and in 4.34% in the older group. Depressive disorders were identified in 0.98% and 2.69%, respectively. Rates of anxiety and depressive disorders were higher among females than males across both age groups [28].

### 2.1. Depression

Depression is one of the most common mental health disorders in the world, with extremely high prevalence among adolescents. Research indicates that depression manifests differently in males and females. While psychological and cultural factors can influence these sex differences, biological factors also play a significant role. For instance, females typically present with depression at an earlier age, report lower quality of life, and exhibit higher rates of comorbidity with anxiety disorders than males who suffer from depression [29].

Researchers have also explored the link between sex differences in the brain and the unique genetic composition of the XY (male) and XX (female) chromosomes. Differences in activation on the X and Y chromosomes result in higher expression of some genes in females. There is some evidence to suggest that the sex chromosome complement may directly affect the size of the cortical and limbic areas related to anxiety and depression [29].

Another factor related to sex differences in depression is related to cortisol levels among males and females. A systematic review and meta-analysis on cortisol found that both male and female patients with depression show higher evening basal cortisol levels compared to healthy individuals, suggesting that poor emotion regulation and stress coping strategies throughout the day may lead to elevated evening cortisol. Additionally, females with depression have higher hair cortisol levels than males with depression, indicating greater cumulative cortisol exposure and prolonged stress in women. Females with depression also exhibit higher cortisol awakening response (CAR) compared to both depressed males and healthy females, which may suggest negative anticipation of the day ahead and poorer sleep quality [30].

Research suggests that the post-pubertal increase in gonadal hormones (estradiol and testosterone) is linked to a higher risk of major depression, particularly in females, whose prevalence is nearly twice that of males [31,32]. This disparity is supported by evidence suggesting that gonadal hormones play a crucial role in mood regulation and susceptibility to depressive disorders [33]. For instance, estradiol enhances HPA sensitivity to stress, acting through estrogen receptor alpha (ERα) in the hypothalamic paraventricular nucleus (PVN), promoting the release of corticotropin-releasing hormone (CRH) and adrenocorticotropic hormone (ACTH), which elevates cortisol levels and contributes to sex differences in stress response [34,35]. Additionally, chronic stress, a known risk factor for major depression, reduces allopregnanolone levels, a metabolite of progesterone, disrupting HPA axis regulation. Since allopregnanolone contributes to glucocorticoid negative feedback, its depletion may heighten female vulnerability to depression [36].

However, biological factors alone do not fully explain the difference; adolescent females also tend to experience more frequent and intense interpersonal stressors, which further contribute to their higher rates of depression compared to males [32].

The significant sex disparity in depression, particularly among adolescents, is a recurring theme in scientific research. The outlook for females is particularly bleak, reflected in higher rates of depression, and aligns with numerous studies on the topic. For instance, a U.S. survey of over 160,000 adolescents highlighted a rise in depression among 12- to 17-year-olds from 2009 to 2019, with a more pronounced increase among females [20]. Similarly, a study of the Stockholm Youth Cohort found that 69.3% of adolescents diagnosed with depression were females, compared to 30.7% males. Notwithstanding, both sexes experienced low psychosocial functioning and additional mental health conditions, though females were more likely to have internalizing disorders, while males had higher rates of externalizing and developmental disorders [37]. Still, outcomes for females appear to be more alarming. For instance, depressive episodes tend to last longer in females. This can be linked to a higher prevalence of physical and sexual abuse, fluctuations in gonadal hormones, and increased episodes of interpersonal stressors among women [38].

### 2.2. Anxiety

Anxiety disorders are one of the most common mood disorders in childhood and adolescence [39]. In 2018, 44% of youth met criteria for an anxiety disorder [40]. This prevalence increases with age [41,42,43], and females are twice as likely to develop an anxiety disorder than males—a gap that widens as adolescents grow older [44,45]. Additionally, anxiety symptoms in females tend to be more severe than in males, particularly among young people [46,47,48]. In this regard, females report more pronounced somatic symptoms, including heightened sensitivity to unpredictable threats, which amplifies anxiety perception [49]. Female adolescents also exhibit higher levels of internalizing symptoms [50,51] and greater sensitivity to daily stressors, especially interpersonal ones [52]. These patterns may provide insight into how anxiety trajectories vary across adolescents and between sexes. In both sexes, a high anxiety trajectory is shaped by depressive symptoms and social connections with peers and teachers. However, only in females, academic competence serves as a predictor of heightened anxiety, with introjected regulation further intensifying this trajectory [53].

Given the many factors contributing to these sex differences, a bio-psycho-social model provides a more comprehensive understanding. Biologically there are functional brain differences between females and males in areas in charge of processing fear and anxiety, such as the prefrontal cortex, hippocampus, and extended amygdala complex [54]. For instance, the amygdala in females reacts with a more intense fear response than in males [55]. Additionally, adolescence is a stage of the life cycle in which significant hormonal changes occur; the levels of gonadal hormones increase significantly compared to previous stages of development, which impacts the mood of adolescents. Furthermore, the importance of hormones in sex differences associated with the prevalence of anxiety disorders has been related to the hormonal fluctuations that occur during the menstrual cycle. In that sense, low levels of gonadal hormones at the end of the luteal phase have been associated with increased anxiety symptoms [46,56]. This may be linked to the modulation of HPA activity by progesterone through its interactions with gamma-aminobutyric acid (GABA) receptors, more specifically with GABA_A_ subtype. Allopregnanolone enhances GABA_A_ receptor expression, strengthening GABAergic inhibition of the HPA axis. Consequently, low progesterone levels may contribute to increased risk of anxiety in females [57]. In addition, hormonal status also influences coping strategies. For instance, females with lower gonadal hormone levels tend to engage more in active coping strategies [15], which has been linked to more adaptive diurnal cortisol patterns in female adolescents [14].

In terms of psychological factors, as previously discussed, adolescence is a period of heightened interpersonal stressors, as social relationships become increasingly important for development. Notably, nearly 80% of the stressors reported by adolescents are linked to interpersonal interactions [41,58]. During this period of cognitive and emotional development, adolescents develop coping strategies for managing daily stressors. This is important, as the use of disengagement coping strategies has been positively associated with an increase in symptoms of stress and anxiety. Notably, this effect was stronger in female adolescents, suggesting that the use of disengagement coping strategies is more harmful for females than for males. In contrast, the use of secondary control coping strategies like cognitive reappraisal or acceptance has been shown to decrease symptoms of anxiety more effectively in females than in males [41,59,60]. Cognitive reappraisal and acceptance play a role in emotion regulation, and their ability to reduce anxiety is likely mediated by their effect on decreasing internalizing symptoms [61].

When considering social factors that may contribute to sex differences in the prevalence of anxiety disorders, assessing the socialization process is key as it can influence the development and acquisition of behaviors and patterns that might increase cognitive and emotional risk of a mood disorder. Several studies have pointed to a possible association between a greater adjustment to the feminine stereotype and anxiety symptoms [62]. Furthermore, stronger identification with traditionally masculine traits such as problem-solving, competitiveness or self-confidence, correlates with increased levels of well-being, perceived control, independence and self-confidence, not only in males, but also in females that identified with these personality traits [46,63,64,65].

While several biological and psychosocial factors, such as gonadal hormones and increased exposure to interpersonal stressors, have been identified as contributing to sex differences in depression and anxiety, the exact mechanisms underlying these disparities remain only partially understood. For instance, it is still unclear how hormonal changes interact with environmental stressors over time, or why some individuals develop emotional disorders while others do not, despite similar exposures. Moreover, because both depression and anxiety are closely linked to suicide, it is essential to explore how these disorders may interact to influence suicide risk, particularly in adolescent populations.

## 3. Depression, Anxiety, and Suicide During Adolescence

Among adolescents, suicide risk is commonly associated with negative emotional states and frequent exposure to stressful life circumstances. Research highlights key risk factors for suicidal behavior in this population, including difficulties in social relationships, such as those with family, romantic partners, or peers, as well as biopsychosocial challenges like depression, substance use, other mental health disorders, and a history of suicidal behavior. Additionally, the four cyclothymic temperaments have been associated with suicidal ideation and suicide attempts [66]. Regarding social factors, challenging academic or work environments, as well as broader factors like social and economic inequality, have also been identified as significant contributors to suicide risk among adolescents [67].

Studies also point to a positive correlation between mental disorders and suicide risk. A systematic review of studies from eight countries focused on identifying a wide range of risk factors for suicidal behaviors in individuals aged 12 to 26 years: the United States, New Zealand, Norway, Australia, Brazil, Canada, Finland, and the United Kingdom. The research found a significant increase in suicide rates among young people with mental disorders [68].

Furthermore, additional risk factors related to sex, gender identity, and sexual orientation have been identified. A literature review on suicide and suicidal behavior identified several risk factors spanning different aspects of an individual’s life, including personal and social dimensions [69]. Key factors include sex, age, depression, anxiety, substance abuse, interpersonal relationships, and bullying. Adolescents, particularly those between the ages of 14 and 16, are among the most affected by this public health issue. The most common risk factors contributing to suicidal behaviors in adolescents are emotional challenges like depression, anxiety, academic stress, and bullying. Many students are vulnerable to peer pressure and often become victims of bullying, which increases their risk of suicide. Individuals experiencing suicidal ideation tend to have lower levels of social support.

Regarding sex differences in suicidal behavior, evidence from 67 analyzed studies indicates that females are almost twice as likely as males to attempt suicide. Conversely, males face a 2.5 times higher risk of dying by suicide compared to females [70]. This highlights that, while suicidal ideation and attempts are more frequent in females, their methods tend to be less lethal overall. These differences underscore the complexity of suicidal behavior and suggest distinct underlying risk factors for each sex [70,71,72].

A systematic review of nonclinical populations aged 12 to 26 in Canada and the United States identified both shared and sex-specific risk factors for suicidal behavior. The review found that females had higher rates of suicide attempts, while males had a greater incidence of suicide deaths. Several risk factors were common across sexes, including bullying, childhood maltreatment, exposure to violence, previous suicidal thoughts and behaviors, family history of mental health disorders, and substance abuse. However, the review also noted differences in how these risks manifested or were weighted by sex, suggesting that while both males and females may experience these adversities, the pathways to suicidal behavior may differ. For example, males may be more influenced by externalizing behaviors and impulsivity, while females may be more affected by internalizing symptoms like depression or interpersonal stress. These findings emphasize the importance of understanding not just which risk factors are present, but how they may impact suicidal behavior differently across sexes [71].

In this vein, a study utilizing the Canadian Vital Statistics Database examined causes of death by analyzing information from death certificates based on International Statistical Classification of Diseases codes. This research provided insights into suicide data for all recorded deaths among Canadians aged 10 and older, revealing a notable and consistent rise in suicide rates among adolescent females aged 10 to 14. This trend suggests increasing distress and/or unhealthy coping mechanisms within this demographic. Contributing factors may include a growing number of school-aged females in Canada reporting symptoms of depression, which are strongly linked to suicide risk. Additionally, shifting gender norms, increased exposure to self-harm content, awareness of more lethal suicide methods, and the unintended normalization of suicide as a response to distress could also be playing a role in these rising rates [72].

Additionally, a study by the Institut de la Statistique du Québec examined data on suicidal thoughts and attempts among 1618 participants aged 13, 15, 17, and 20 years. The findings revealed increased rates of suicidal ideation and attempts, particularly among females. These sex disparities likely reflect multiple underlying factors. For instance, females tend to experience higher rates of depression, which is strongly linked to suicidal behavior, whereas males may well face greater social stigma surrounding emotional expression and help-seeking, which can mask their distress or discourage them from reporting suicidal thoughts. These findings highlight the complex and gendered nature of mental health and suicide risk during adolescence [73].

As a result, the scientific literature supports a strong relationship between suicide and mental health disorders, especially anxiety and depression [68,69,73]. Given the link between depression, anxiety, and suicide, designing timely and effective interventions for adolescents is of paramount importance in stemming the tide of increasing suicidal thoughts, behavior, and attempts.

## 4. Current Interventions

Given the significant differences in how depression and anxiety affect males and females, it is essential to examine whether these differences are adequately addressed when evaluating treatment options and mental health interventions. Two critical questions emerge: Are current approaches effectively targeting these gender disparities? And how accessible are current treatments to those who need them?

Research has highlighted significant sex differences in the absorption and distribution of antidepressants, and females typically respond more favorably to selective serotonin reuptake inhibitors (SSRIs) than tricyclic (TCAs) antidepressants [74]. SSRIs modulate estrogen synthesis/signaling [75], and the effects of SSRIs are enhanced during the luteal phase, when estrogen is lowest [76]. While pharmacotherapy takes biological sex differences into account, the way psychosocial factors play a role in treatment outcomes should also be the focus of additional study.

Cognitive behavioral therapy (CBT) and interpersonal therapy (IT) present promising results in decreasing anxiety and depressive symptoms in youth [41,77,78]. At present, CBT has the most convincing body of evidence supporting the treatment of anxiety, and it is the only intervention for which controlled experiments have shown its efficacy. CBT is comprised of four strategies: exposure, contingency management, cognitive strategies, and modeling [79,80], which are applied based on the anxiety disorder the patient presents. Evidence regarding sex differences in CBT outcomes for treating symptoms of anxiety is currently limited [81]. More in-depth analysis is needed, since some factors appear to be more effective in reducing anxiety symptoms in children and adolescents based on sex. One instance is related to the patient’s coping strategies. As noted above, disengagement coping strategies, such as attempting to distance oneself from the stressor or the emotion derived from the stressor, are often related to higher anxiety and depression symptoms, while primary and secondary control coping strategies help reduce the impact stressful situations have. Primary control strategies include actively changing the emotional reaction to the stressor, while secondary control strategies employ more complex cognitive strategies to adapt to the stressor, such as reappraisal or acceptance. Interventions that aim to increase the use of primary and secondary coping strategies while reducing the use of disengagement strategies are particularly valuable for youth, as adolescence is a critical period for developing effective coping mechanisms [41]. Research indicates that interventions that promote primary and secondary coping strategies are particularly beneficial for females as the impact of these coping strategies on mental health is often more powerful than the observed effect in males [41,43]. Further research on sex-specific interventions is essential, as these differences have been shown to be particularly relevant to how patients deal with and attempt to recover from depressive disorders.

Cultural norms and gender expectations significantly influence not only the decision to seek treatment but also how mental health symptoms are expressed and interpreted, factors which directly impact diagnosis. For example, societal expectations often associate masculinity with strength, stoicism, and emotional restraint, making expressions of sadness or vulnerability socially unacceptable for many males. These norms discourage emotional openness and delay or prevent individuals, particularly adolescent boys, from seeking help or even recognizing their own symptoms as signs of mental distress.

A qualitative study using narrative–biographical interviews found that most participants believed masculine norms influenced their reluctance to seek help. Many expressed discomfort with discussing emotions or crying and preferred to manage their health privately rather than consult professionals [82]. Other studies also confirm that traditional masculinity norms reduces the likelihood of help-seeking behavior, thereby reinforcing gender-based social constructs [83]. These expectations, which are internalized from early childhood and adolescence, create substantial barriers to accessing mental health care, especially for adolescents, who are still developing their identity [84].

Sex differences in help-seeking persist through adulthood. U.S. data from 2022 showed that 56.9% of adult females received mental health treatment compared to only 41.6% of adult males [85]. Though this data reflects adult populations, it underscores long-standing sex disparities that are rooted early in life and continue into adulthood. Addressing the cultural and societal barriers that discourage help-seeking is crucial to improving early detection and access to care, especially for adolescents.

This is particularly relevant when comparing adolescents to adults. While adults may have developed more self-awareness, emotional vocabulary, and agency to seek help, adolescents are often still navigating societal expectations, peer pressure, and family influence, all of which can distort the presentation of depressive symptoms. In adolescence, symptoms may be more likely to present as irritability, anger, or somatic complaints, rather than the more “classic” adult symptoms like persistent sadness or withdrawal [86]. These alternative presentations, shaped in part by gender norms and social expectations, make accurate diagnosis more challenging.

Given the strong influence of cultural norms and social constructs on emotional expression and help-seeking behavior, it is unsurprising that diagnosing depression in adolescents is particularly complex. Unlike adults, adolescents face additional developmental challenges, including identity formation and peer conformity, which further complicate symptom recognition both internally and by health care providers. Early diagnosis is essential, as prognosis significantly improves with prompt treatment. Common treatment approaches include psychotherapy (especially cognitive behavioral therapy and interpersonal therapy), pharmacotherapy, and the involvement of family and psychoeducation [87,88]. For effective outcomes, it is critical that treatment considers the adolescent’s broader cultural, familial, and social environment.

Beyond the differences related to cultural and societal norms, treatment recommendations for mild and moderate to severe depression among teenagers between 12 and 18 years old also vary slightly based on the type of depression. Psychotherapy, such as online or group-based cognitive behavioral therapy, group interpersonal psychotherapy, and group non-directive supportive therapy, are recommended for mild depression. Likewise, given the important role family plays in treatment, attachment-based family therapy is also recommended. Medication, however, is not recommended as an initial treatment option. Meanwhile, treatment for moderate to severe depression also includes family therapy (attachment-based or systemic), individual CBT, psychodynamic psychotherapy, and interpersonal psychotherapy (IT). Unlike treatment for mild depression, combined therapy (a combination of both medication and psychological therapy) is considered a viable option for moderate to severe depression [89]. Concerning sex differences, some evidence suggests that females with mild-to-moderate depression respond less effectively to CBT, compared to males and females with more severe depression [90]. Unfortunately, most studies conducted with adolescents do not provide a clear analysis of sex differences in treatment outcomes.

However, many young people still lack sufficient access to mental health information, underscoring the need for greater awareness, more education, and additional interventions to improve their mental well-being. Educational centers can play a pivotal role in this effort by promoting a holistic approach where schools collaborate with the community, students, families, and staff to foster better mental health support [20]. Since adolescents spend more active time at school than at home, schools are a critical component of the mental health system and vital for identifying and addressing mental health issues among young people [91]. The school environment is increasingly important for detecting risks and implementing policies that help to reduce and mitigate these risks. Empirical evidence shows that the educational setting is appropriate and optimal for implementing mental health programs related to suicide risk [92]. Demystifying mental health and offering suicide prevention interventions in schools makes educational centers safer while promoting mental well-being [93].

Among the strategies used to achieve these goals are sensitivity training, information dissemination, and therapeutic interventions. One effective strategy is training school staff to act in cases of suicidal risk, also called gatekeeper training. The objective of training gatekeepers is to develop skills related to communication, identifying suicidal behavior, and providing emotional support [79]. Teachers play a fundamental role in these programs, given their daily contact with the adolescents. Additionally, teachers often have hands-on experience with students at risk of or who have attempted suicide, allowing them to act as agents of prevention. However, it is important to provide adequate support through active training programs that includes role-playing, where teachers learn how to respond to at-risk students and refer them to appropriate mental health centers [80].

Despite numerous studies highlighting sex differences in depression, anxiety, and suicide, these differences are generally not taken into consideration when designing gatekeeper programs. However, research suggests that behavioral responses to such training do indeed vary by sex. For instance, females are more predisposed to intervene and demonstrate greater knowledge about suicide compared to males [94]. In a study involving 231 U.S. students, females were more receptive to gatekeeper training and showed significant improvement in knowledge and skills after participating [95].

## 5. Conclusions and Future Perspectives

Depression and anxiety are highly correlated to suicide and are a critical public and mental health issue. While sex differences in these conditions are widely accepted in the scientific literature, interventions are generally aimed at differentiating only between adolescents, adults, and the elderly. In this context, it is crucial to enhance research efforts to clearly identify sex differences in risk factors, particularly during adolescence, a high-risk stage, and to comprehensively monitor variations in treatment outcomes. These factors must also consider biological parameters, such as hormonal fluctuations throughout the menstrual cycle and their epigenetic effects on other hormones like cortisol, as well as medication and mood. In this regard, a recent study identified altered DNA methylation in patients with anxiety disorders at 148 CpG sites in genes associated with anxiety, anxiety-related disorders, and brain function. Furthermore, DNA methylation changes were observed in response to CBT at four CpG sites from baseline to the six-month follow-up [96]. These findings underscore the potential of epigenetic markers in refining personalized treatments for mood disorders, enabling more targeted and effective therapeutic strategies. Similarly, social factors, especially those related to gender roles and exposure to traumatic or violent events, are of particular interest. Additionally, from a psychological perspective, stress coping mechanisms, personality traits, and mood are all factors that can contribute to a broader understanding of sex differences in vulnerability and protective factors. This comprehensive approach will enable the development of more effective, personalized interventions.

Future studies should further investigate sex differences to strengthen the evidence on their impact on intervention responses for mood disorder symptoms and suicide risk. Regarding CBT interventions, researchers should examine whether sex differences influence the benefits of cognitive reappraisal-based interventions compared to behavioral activation and exposure therapy. Additionally, the role of peer influence in therapy effectiveness should be explored, assessing whether group-based CBT enhances outcomes more in females, who may rely more on social support, compared to males, who might prefer individualized approaches. Concerning pharmacological therapies, research should focus on monitoring the persistence of treatment effects over time, evaluating whether sex differences impact relapse rates or sustained improvement. Further investigation is needed into dose optimization, examining sex-based variations in SSRI metabolism, and adjusting pharmacokinetics accordingly. Additionally, studies should explore whether sex differences influence the frequency and severity of adverse effects, such as weight changes, fatigue, or emotional blunting. Finally, in school-based interventions, future research should assess differences in targeted coping skill training, identifying which programs are most effective for females and males. Additionally, studies should explore whether mentorship and gatekeeper programs differentially reduce symptoms of anxiety and depression based on sex, refining intervention strategies to better support adolescent mental health.

Interventions based on an individual’s unique genetic, biological, and environmental factors, focusing on the specific needs of each person, can lead to more precise and effective care. At the same time, implementing comprehensive prevention approaches that focus on identifying protective factors related to social relationships is equally important. These approaches include promoting stable relationships during childhood, fostering connectedness among teenagers with their parents, teachers, and friends, and creating secure environments at home and in schools [97].
